# Quantifying the Decisional Satisfaction to Accept or Reject the Human Papillomavirus (HPV) Vaccine: A Preference for Cervical Cancer Prevention

**DOI:** 10.1371/journal.pone.0088493

**Published:** 2014-02-14

**Authors:** Diane M. Harper, Billy B. Irons, Natalie M. Alexander, Johanna C. Comes, Melissa S. Smith, Melinda A. Heutinck, Sandra M. Handley, Debra A. Ahern

**Affiliations:** 1 Department of Community and Family Medicine, University of Missouri Kansas City School of Medicine, Kansas City, Missouri, United States of America; 2 Department of Obstetrics and Gynecology, University of Missouri Kansas City School of Medicine, Kansas City, Missouri, United States of America; 3 Department of Biomedical and Health Informatics University of Missouri Kansas City School of Medicine, Kansas City, Missouri, United States of America; 4 Department of Family Medicine, Kansas City University of Medicine and Biosciences, Kansas City, Missouri, United States of America; 5 Student Health and Wellness, University of Missouri-Kansas City, Kansas City, Missouri, United States of America; The Catalan Institute of Oncology (ICO), Spain

## Abstract

**Objective:**

Only a portion of the US population is willing to consider HPV vaccination to date. The primary aim of this study is to determine the decisional satisfaction associated with HPV vaccination.

**Study Design:**

This is a prospective survey conducted at an urban college where women 18–26 years old completed a decisional satisfaction survey about their HPV vaccine experience.

**Results:**

Regardless of the decision to accept or reject HPV vaccination, the decisional satisfaction was very high (mean 5-item score = 21.2 (SD 3.8)). Women without HPV vaccination were decisionally neutral significantly more often than those already vaccinated; 22% were decisionally neutral for the option to accept HPV vaccination at that visit. Cervical cancer prevention was preferred significantly more often than genital wart prevention in all analyses.

**Conclusions:**

Targeting those who are decisionally neutral about HPV vaccination may result in a higher uptake of HPV vaccination.

## Introduction

Many women’s health screenings including cancer prevention involve patient education, patient participation and shared decision making. In order to reach a high-quality decision, the benefits and harms of the alternative options must be clearly discussed. More importantly, the choice depends on how patients value benefits versus harms. The eventual goal is a strong match between the chosen option and the features that matter most to the informed patient [1].

Much of human papillomavirus (HPV) vaccination in the US has been presented through the eyes of public health with an overriding sense of community obligation to be vaccinated for whatever herd immunity may occur [2], [3]. Healthy People 2020 aims for very high population vaccination rates [4], but recent surveys indicate only a portion of the population is willing to consider HPV vaccination [5]. While the need for understanding the social and decisional psychology of vaccine uptake is acknowledged [6], little work has studied these concepts in young women concerning their decision to be vaccinated against HPV infection, and whether the option of choosing between two different prophylactic HPV vaccines was important to them. Likewise, there has been little documentation of decisional conflict or decisional regret during or after the HPV vaccine decision making process, as there has been for influenza vaccination [7], [8].

The primary aim of this paper is to quantify the satisfaction of the decision to receive at least one dose of HPV vaccine among young adult women. The secondary aim was to determine which demographic and medical history items influenced the value of cervical cancer prevention vs. genital wart prevention followed by choice of HPV vaccine.

## Methods

This prospective study was approved by the University of Missouri Kansas City (UMKC) Social Sciences Adult Institutional Review Board (SSIRB) (#SS10-56X) and is part of a larger study on women’s preferences for HPV vaccination.

All women, 18–26 years old, being seen at UMKC Student Health and Wellness, an urban college health service, were offered the opportunity to participate in the survey between January 2011 and August 2012. All members of the health care staff were educated on the purpose of the study; and provided scripts to introduce it at check-in. Women were only allowed to complete the survey once, and they self-reported demographic and medical history. For those who had received an HPV vaccine, we did not ask number or timing of doses. The study was open to both those with prior HPV vaccination and those who had not yet started the series. Women completed the survey prior to being seen by the health care provider, and could ask questions about HPV vaccination during the visit.

The cervical cancer prevention education information was constructed in a format similar to a decision aid [1]. It provided information on the benefits and harms associated with cervical cancer screening programs, with HPV vaccination alone (specifying the referenced differences between the two HPV vaccines), and with the combination of HPV vaccination and cervical cancer screening [9].

The decisional satisfaction scale was adapted for cervical cancer prevention from the original Holmes-Rovner model [10]. It is a unidirectional six item set of questions rated on a 5 point Likert scale (1 = strongly disagree, 5 = strongly agree). Higher scores indicate a higher satisfaction with the decision. Individual responses and a 5-item summary score were the outcomes of interest. The item assessing “expected action” was analyzed only for those who had not made a choice to receive at least one dose of HPV vaccine prior to the survey.

Three items were included in the survey modeled on the O’Connor’s decisional conflict scale [11] that queried the perceived value of preventing different HPV-associated diseases: cervical cancer and genital warts. A final item presented a choice trading off between the two vaccines.

Statistics: Descriptive statistics included means testing with Student’s t-test for significance testing. Chi-square comparison and Fisher’s exact testing were used for ratios as appropriate and p-values less than 0.05 were considered significant [12].

## Results

Over five academic terms, 10,562 patients (male and female ages 16–62 years) were seen at the Student Health and Wellness clinic. Of these, 306 women without prior HPV vaccination and 299 vaccinated women agreed to participate in the survey. The average age did not differ between the two groups, but for the vaccinated women the average age of first intercourse was significantly lower and the average number of lifetime sexual partners was significantly higher (Table 1). Over 90% of the participants had never been pregnant. Significantly more white and fewer Asian women were vaccinated than not. Hormonal contraceptive use did not differ between vaccinated and unvaccinated groups, but those who were vaccinated used condoms significantly more than those not vaccinated.

**Table 1:** Demographic Characteristics of Women with and without HPV Vaccination.

	No HPV Vaccine			At least one dose				
	N	mean	SD		N	mean	SD	p-value
Age	291	21.6	2.3		289	21.3	2.34	NS
Age at first intercourse	229	17.4	1.98		249	16.87	1.98	0.003
Number of lifetime sexual partners	267	3.3	4.66		269	4.76	5.81	0.001
	**N**	**%**			**N**	**%**		
Gravidity								
n = 0	250	90.9			250	90.3		NS
n≥0	25	9.1			27	9.7		
Race/Ethnicity								
White	172	57.1			200	67.8		0.007
Black	61	20.3			47	15.9		NS
Hispanic	13	4.3			10	3.4		NS
Asian	35	11.6			17	5.8		0.011
Other	20	6.6			21	7.1		NS
Contraceptive Use								
No	164	56.0			141	49.1		NS
Yes	128	43.7			145	50.5		
Condom Use								
No	126	44.5			86	30.9		0.001
Yes	156	55.1			187	67.3		
Sometimes	1	0.4			5	1.8		
History of Pap test*								
No	44	23.9			8	4.9		<0.001
Yes	140	76.1			156	95.1		
History of Abnormal Pap test*								
No	146	78.9			118	71.5		NS
Yes	39	21.1			47	28.5		
History of HPV Infection*								
No	172	93.0			137	83.0		0.004
Yes	13	7.0			28	17.0		
History of Colposcopy*								
No	162	87.6			137	83.0		NS
Yes	23	12.4			28	17.0		
History of Genital Warts								
No	288	98.3			277	96.5		NS
Yes	5	1.7			10	3.5		
History of Other STIs								
No	268	91.5			251	87.2		NS
Yes	25	8.5			37	12.8		

*among those 21 years and older.

Student’s t-test was used to compare continuous variables. Chi-square testing and Fisher’s exact test was used to compare proportions.

Among the women 21 years and older, age-appropriate for cervical cancer screening, 24% of those without any doses of HPV vaccine had never had a Pap test, significantly higher than those with at least one dose of HPV vaccine. There were similar proportions of women with abnormal Pap tests and who underwent colposcopy in both the vaccinated and unvaccinated groups. Significantly more women with prior HPV infections had been vaccinated. A past history of genital warts or of other sexually transmitted infections (STIs) was equally distributed between the vaccinated and unvaccinated groups.

### Satisfaction with Decision to Receive at Least One Dose of HPV Vaccine

The decisional satisfaction scores on the five item survey were high regardless of HPV vaccination status (Table 2) indicating that women were comfortable with their decision to date of accepting or rejecting HPV vaccination. For instance, satisfaction scores for “This decision is best for me personally” were equally high for both acceptors and rejecters of HPV vaccination. The decisional satisfaction scores from the other four qualities of the decision, while high for both groups, were significantly higher for those accepting rather than rejecting HPV vaccination indicating a high polarization towards vaccination among those choosing vaccination.

**Table 2:** Decisional Satisfaction.

	No HPV Vaccination		At least one Dose of HPV Vaccine		
	N	Mean (SD)	N	Mean (SD)	p-value
I am informed about the cervical cancer prevention options	303	4.0 (0.9)	297	4.2 (1.0)	**0.021**
This decision is best for me personally	303	4.1 (0.9)	296	4.2 (1.0)	NS
This decision is consistent with my personal values	302	4.2 (0.8)	290	4.4 (0.9)	**0.001**
I am satisfied that the decision was mine to make	300	4.2 (0.9)	287	4.4 (0.8)	**0.009**
I am satisfied with my decision	301	4.1 (0.9)	289	4.4 (0.9)	**<0.001**
5-item Summary Score	300	20.7 (3.5)	220	21.8 (4.0)	**<0.001**
I expect to be vaccinated before leaving the office	301	2.1 (1.0)			
**Decisional Neutrality (score = 3)**	**N**	**%**	**N**	**%**	
I am informed about the cervical cancer prevention options	30	9.9	25	8.4	NS
This decision is best for me personally	58	19.1	35	11.8	**0.013**
This decision is consistent with my personal values	50	16.6	20	6.9	**<0.001**
I am satisfied that the decision was mine to make	35	11.7	14	4.9	**0.003**
I am satisfied with my decision	49	16.3	17	5.9	**<0.001**
I expect to be vaccinated before leaving the office	65	21.6%			

Bold indicates statistical significance.

Student’s t-test was used to compare continuous variables. Chi-square testing and Fisher’s exact test was used to compare proportions.

The “expectation to be vaccinated at this visit” choice showed low satisfaction scores for those not yet vaccinated (mean 2.1 (SD 1.0)), meaning that there was substantial disagreement with the intent to start HPV vaccination among those not yet vaccinated.

A decisional satisfaction score of 3 indicates neutrality and potential decisional conflict where certainty about choice does not yet exist. The highest decisional neutrality occurred among those without HPV vaccination regarding expectations of being vaccinated before leaving the office (22%). The lowest decisional neutrality occurred for the item “I am informed about the cervical cancer prevention options” indicating that only 9% of women regardless of HPV vaccine status were uncertain about their cervical cancer prevention options.

Whereas a certain amount of decisional neutrality was evident among all women regardless of HPV vaccine status, women with no prior HPV vaccination had significantly greater decisional neutrality on the remaining four qualities of decisional satisfaction than those with at least one HPV vaccine dose.

### Perceived Value of Prevention of Different HPV Associated Diseases

Women with diverse medical histories ranked the currently perceived value of preventing different HPV associated diseases differently (Table 3). Cervical cancer prevention was significantly more important than genital wart protection among all women overall (mean 4.5 (SD 0.8) vs. 4.3 (SD 1.0), P = 0.014). Where there were differences within medical histories, the women at least risk for cervical cancer valued cervical cancer prevention significantly more highly than genital wart prevention: women with a history of at least one dose of HPV vaccine; with no prior STIs; with at least one prior Pap test, but with no prior cytologic abnormalities; with no prior HPV infections and no prior colposcopies.

**Table 3:** Value of Disease Prevention by Medical History.

	Cervical Cancer Prevention			Genital Wart Prevention			
	N	Mean (SD)	p-value	N	Mean (SD)	p-value	P-Value*
Prior HPV Vaccination							
No	295	4.2 (0.9)	**<0.001**	295	4.1 (1.1)	**<0.001**	
Yes	284	4.7 (0.6)		284	4.6 (0.8)		**0.041**
Prior STI							
No	509	4.4 (0.8)	**0.002**	509	4.3 (1.0)	**<0.001**	**0.003**
Yes	59	4.8 (0.5)		59	4.8 (0.6)		
**Women ≥21 years**							
Prior Pap testing							
No	51	4.1 (1.0)	**<0.001**	51	4.0 (1.3)	**0.006**	
Yes	288	4.5 (0.7)		288	4.4 (0.9)		**0.003**
Prior abnormal Pap test							
No	83	4.4 (0.8)	**0.007**	83	4.2 (1.0)	**0.002**	**0.001**
Yes	258	4.7 (0.6)		258	4.6 (0.8)		
Prior HPV Infection							
No	40	4.4 (0.8)	**0.006**	40	4.3 (1.0)	**0.001**	**0.001**
Yes	301	4.8 (0.5)		301	4.8 (0.5)		
Prior Colposcopy							
No	51	4.4 (0.8)	**0.041**	51	4.3 (1.0)	NS	**0.005**
Yes	290	4.7 (0.7)		290	4.5 (0.8)		

Bold indicates statistical significance.

*P-value refers to the Student’s t-test comparison of importance between cervical cancer vs. genital wart prevention by medical history item (e.g. among women with at least one dose of HPV vaccine, cervical cancer prevention is significantly more important than genital wart prevention, 4.7 vs. 4.6, p = 0.041).

Women who experienced HPV related disease had higher preferences for cervical cancer and genital wart prevention than inexperienced women. For instance, women with at least one dose of HPV vaccine valued cervical cancer and genital wart prevention more than women with no vaccination (cervical cancer: mean 4.7 (SD 0.6) vs. mean 4.2 (SD 0.9), p<0.001); genital warts: mean 4.6 (SD 0.8) vs. mean 4.1 (SD 1.1) p<0.001). Similarly, women with a prior STI valued cervical cancer and genital wart prevention more than women without a prior STI. Among women 21 years and older, those with a prior Pap, with a prior abnormal Pap and a prior HPV infection valued cervical cancer and genital wart prevention more than those women without the respective medical histories. A past colposcopic experience only enhanced the women’s value of cervical cancer prevention, not genital wart prevention.

Genital wart prevention was never more highly valued than cervical cancer prevention in any sub-population. Significantly more women would trade off genital wart prevention for more cervical cancer prevention (57% (312/547) vs. 43% (235/547), p<0.001); this preference remained when stratifying by prior HPV vaccine receipt (no vaccine: 57% (160/279) vs. 43% (119/279), p<0.001; vaccine: 56% (152/268) vs. 44% (116/268), p = 0.002).

When asked to make a choice between vaccines, significantly more women chose HPV2 over HPV4 (61% (325/537) vs. 39% (212/537), p<0.001, Table 4). This preference was significantly more pronounced among those 21 years and older, with no pregnancies, not using hormonal contraceptives but using condoms, not having received any HPV vaccine doses, with no prior Pap tests, no prior abnormal cytologies if a Pap test had been done, no prior HPV infections, no history of colposcopy and no history of other STIs. Among those women already having received at least one dose of HPV vaccine, significantly more women would have chosen HPV2 over HPV4 had they been given the choice initially (54% (145/267) vs. 46% (122/267), p<0.05).

**Table 4:** HPV Vaccine Choice by Medical History.

	Prefer HPV2†		Prefer HPV4‡		
	N	%	N	%	p-value
Age					
<21 years	114	56.2	89	43.8	**0.013**
≥21 years	205	62.5	123	37.5	**<0.001**
Gravidity					
n = 0	282	61.0	180	39.0	**<0.001**
n≥0	21	48.8	22	51.2	NS
Contraceptive Use					
No	171	61.1	109	38.9	**<0.001**
Yes	144	58.1	104	41.9	NS
Condom Use					
No	115	59.6	78	40.4	**<0.001**
Yes	190	60.7	123	39.3	**<0.001**
History of HPV Vaccine					
No	183	65.8	95	34.2	**<0.001**
Yes	145	54.3	122	45.7	**<0.05**
History of Genital Warts					
No	309	59.9	207	40.1	**<0.001**
Yes	8	61.5	5	38.5	**<0.001**
History of Other STIs					
No	287	65.7	186	42.6	**<0.001**
Yes	30	53.6	26	46.4	NS
History of Pap test*					
No	41	85.4	7	14.6	**<0.001**
Yes	155	57.4	115	42.6	**<0.001**
History of Abnormal Cytology*					
No	158	65.6	83	34.4	**<0.001**
Yes	40	50.6	39	49.4	NS
History of HPV Infection*					
No	180	63.6	103	36.4	**<0.001**
Yes	18	48.6	19	51.4	NS
History of Colposcopy*					
No	174	63.7	99	36.3	**<0.001**
Yes	24	51.1	23	48.9	NS

*Among women ≥21 years old.

† HPV2 means bivalent vaccine, Cervarix®.

‡ HPV4 means quadrivalent vaccine, Gardasil® or Silgard®.

Chi-square or Fisher’s exact testing were used to compare proportions.

Bold font indicates statistical significance.

## Comment

Our work is the first to explore decisional satisfaction with HPV vaccination. Some have studied how normative messaging from peers, family and health professionals influence HPV vaccine choice [13], [14]; others have studied the Health Belief Model where the decision to vaccinate is a balance between the perceived risk of disease severity including its health and social consequences as well as the benefits/harms of vaccination [15], [16].

Recently discrete choice experimentation (DCE) has provided a health economics metric to understand how adults trade off risks and benefits among competing options for similar health care outcomes [17], [18], such as HPV vaccination and Pap testing to prevent cervical cancer, in terms of willingness to pay or willingness to trade [19].

These theoretical models have resulted in using strong health care provider messaging for vaccination, promoting the commonness of HPV infections associated with multi-organ cancers, and emphasizing no out-of–pocket expenses as motivators to increase HPV vaccination rates. However, these strategies have not led to an increase in uptake, but rather a plateauing of those receiving HPV vaccinations [5].

Decisional satisfaction offers the perspective of determining which set of young women are still neutral about their HPV vaccine choice; and hence more likely to be open to more discussion about how the benefits and risks of HPV vaccination play into their value for cervical cancer prevention. While Healthy People 2020 anticipates 80% vaccination of the young adult women during their adolescence, the current trends support a large portion that will still have the opportunity to make a HPV vaccination decision as young adult women. Increased vaccination may be more likely if this decisionally neutral set of young women is targeted.

Women with a clear sense of what their personal value of HPV vaccination was (accept/reject) were highly satisfied with their decision. The women who have had at least one dose of HPV vaccine who expressed neutral satisfaction with their decision to be vaccinated may be indicating decisional regret about a decision made for them at an earlier time. Future research will use decisional conflict and decisional regret scales to attempt to understand these values [11], [20]. In addition, we intend to study the decisional conflict surrounding continuing the second and third doses in a timely manner among young women who initiate HPV vaccination.

Our work shows that young women have greater value for cervical cancer prevention than for genital wart prevention. This was also seen in Oteng’s work among adult women who were willing to trade genital wart protection for cervical cancer protection [19]. Translating these values into vaccine choices has depended on the level of information provided about the vaccines. While Oteng’s work clearly showed the stronger preference for cervical cancer prevention, the attributes offered about the vaccines ignored the additional cervical cancer prevention associated with HPV2, leading to the more frequent choice of HPV4 than HPV 2 in that work. Our work presented the cross protection and duration of efficacy data available for both HPV4 and HPV2 in addition to the regulatory licensure claims. This information most likely facilitated the consistent alignment of preference for more cervical cancer prevention with the choice of HPV2 over HPV4. We should note, though, that having the two options for HPV infection prevention is valuable to young women, as there was not an absolute choice for one vaccine over the other.

Limitations: Our work is limited by several factors. In the time course of making a medical decision, the decision aid (or some degree of information about the medical issue) is presented followed by a survey of the satisfaction associated with the choice made. In our work half the population had already made the choice to receive at least one dose of HPV vaccine. This limitation offers a potential hypothesis-generating benefit, though, in that the decisional neutrality expressed might provide insight into decisional regret they may be experiencing up to five years after initial vaccination.

Within the decisional framework of HPV vaccination, we did not offer the choice of no HPV vaccination; therefore, we do not know how an option of no vaccination would change these results.

Separately, while our study population resembled most young adult HPV studies in that the number of lifetime sexual partners and age at first intercourse is historically similar [21]–[25], the balanced proportions of previously vaccinated vs. unvaccinated women, while occurring by chance, does not reflect the young adult female HPV vaccination rate [26].

Finally, the developmental capacity to make medical decisions is recognized to be more complicated in adults than in adolescents [27]. While these results are likely to be representative of young adults making their HPV related decisions, these results may not be applicable to adolescents or parents making decisions for pre-pubescent youth.

## Conclusions

Targeting young adult females who are decisionally neutral about HPV vaccination may be a more direct method of increasing HPV vaccination rates in a targeted population than spending resources on those already highly satisfied with their decision not to vaccinate.
